# Establishing a peer advisory board in a mental health ethics research group – challenges, benefits, facilitators and lessons learned

**DOI:** 10.3389/fpsyt.2025.1516996

**Published:** 2025-02-28

**Authors:** Mirjam Faissner, Esther Braun, Simone Agnes Efkemann, Anne-Sophie Gaillard, Iris Haferkemper, Christin Hempeler, Imke Heuer, Ursula Lux, Sarah Potthoff, Matthé Scholten, Sylvia Spiegel, Christina Stefaniak, Madeleine Thesing, Anna Werning, Jakov Gather

**Affiliations:** ^1^ Institute of the History of Medicine and Ethics in Medicine, Charité - Universitätsmedizin Berlin, Berlin, Germany; ^2^ Department of Psychiatry, Psychotherapy and Preventive Medicine, LWL University Hospital, Ruhr University Bochum, Bochum, Germany; ^3^ Junior Professorship for Medical Ethics with a Focus on Digitization, Faculty of Health Sciences Brandenburg, University of Potsdam, Potsdam, Germany; ^4^ Institute for Medical Ethics and History of Medicine, Ruhr University Bochum, Bochum, Germany; ^5^ Peer Advisory Board, BMBF Research Group SALUS, Ruhr University Bochum, Bochum, Germany; ^6^ Institute for Ethics, History and Theories of Medicine, University Münster, Münster, Germany

**Keywords:** participatory research, service user involvement, medical ethics, psychiatry, consumer/survivor/ex-patient movement, experiential knowledge

## Abstract

While participatory methods are regarded as beneficial in many areas of psychiatric research, they are still rarely considered in the field of mental health ethics. Yet, there are several epistemic and ethical reasons why participatory research is particularly important in this field, such as the high relevance of experiential knowledge for ethical analyses. In this article, we report our experiences with establishing a peer advisory board for an existing mental health ethics research group. We demonstrate how a peer advisory board can provide low-threshold opportunities for various forms of participation, which can occur simultaneously within one research project. We first describe how we established the peer advisory board and explain its structure. We then give an overview of several research projects that involved various forms of participation by members of the peer advisory board, such as the development of a template for a psychiatric advance directive, the co-writing of articles, and the organization of scientific events. We discuss the challenges, benefits, and facilitators of a peer advisory board from our different vantage points as service users, relatives, clinicians, and researchers. Challenges included organizational barriers such as time constraints and rigid bureaucratic structures within academic institutions and funding bodies as well as the persistence of power imbalances between members of the research group and the peer advisory board. Benefits included the opportunity for personal development and capacity building among both peer advisory board members and members of the research group, and the multiplication of research results among the relevant communities. Based on a reflection on our own experiences, we argue that participatory research in mental health ethics is not only ethically and epistemically desirable but also practically feasible. We close by formulating several lessons learned from our experiences.

## 1 Introduction

Mental health ethics is an interdisciplinary field of research concerned with current practices and policies in mental healthcare. It approaches controversial issues in mental healthcare, such as the meaning and assessment of decision-making capacity or the use of coercive measures, from a normative perspective (see, e.g., Helmchen and Gather^1^ for an overview of central topics in the field). Mental health ethics uses philosophical analysis to evaluate and weigh arguments for and against actions or policies to determine whether such actions or policies are ethically defensible. Central principles in mental health ethics, and medical ethics more generally, are the principles of respect for patient or service user autonomy, beneficence, non-maleficence, and justice (1). These are also recognized in the European Psychiatric Association's code of ethics (2). Often, mental health ethics also combines philosophical research with empirical research that is intended to inform ethical analyses.

Many researchers, service user and relatives’ groups, members of the consumer/survivor/ex-patient (c/s/x) movement, and people involved with the Mad Pride movement stress the importance of participatory approaches in psychiatric research (3–5). The field of mental health ethics, however, has rarely been discussed as a relevant site for participatory research so far. Yet, participatory research in mental health ethics is urgently needed for several reasons.

Historically, people with mental health conditions have been socially marginalized, and the concerns they have voiced with respect to psychiatric practices have been systematically disregarded (6–8). As demonstrated in the report of the Lancet Commission on ending stigma and discrimination in mental health, these forms of marginalization still persist today and people with mental health conditions around the globe continue to face pervasive forms of stigma and discrimination (9). Moreover, mental healthcare is marked by profound power asymmetries, especially in relation to the prerogative of mental health professionals to diagnose people with mental health conditions and to submit them to psychiatric treatment against their will (10). Ethical analyses that fail to appropriately consider the perspectives of mental health service users are prone to explicitly or implicitly perpetuate or even reinforce societal mental health stigma and institutional power asymmetries. The dictum “Nothing about us without us” that posits service users’ involvement as a question of social justice and human rights should accordingly resonate especially strongly with mental health ethicists.

Beyond being a matter of social justice, involving service users in mental health ethics research is also warranted from an epistemic perspective, i.e., based on considerations regarding how the relevant knowledge can be generated. Mental health ethicists often take a bottom-up approach, where they start by exploring the often-intricate details of a practice or policy and the experiences and views of stakeholders based on empirical research. In a second step, they evaluate and weigh these views against each other from a normative perspective to determine whether or not the practice or policy is ethically defensible (11). In this process, experiential knowledge is highly relevant. It involves phenomenological knowledge about what it is like to experience mental distress, receive psychotherapy or be subjected to coercive measures, emotional knowledge about the affective consequences of psychiatric interventions, and practical knowledge about their immediate, short-term and long-term effects. Importantly, people with experiential knowledge can contribute a valuable long-term perspective that goes beyond a snapshot of a particular intervention or experience.

One influential argument that supports the relevance of experiential knowledge for mental health ethics research comes from standpoint theory (12). Standpoint theory builds on the idea that a person’s social position shapes what they can know. More specifically, it assumes that members of marginalized social groups can acquire privileged epistemic access to knowledge domains related to their oppression (13). This social position is best understood as intersectional (14), meaning that it is simultaneously conditioned by multiple social categories, including a person’s mental health, gender, racialization, sexual orientation, class and ability (15). Communities affected by social oppression often develop a nuanced and differentiated understanding of their experiences of oppression, which helps them to navigate and survive these conditions (16). Since service users have an epistemic privilege regarding knowledge domains related to mental health and mental healthcare services, integrating their perspectives is crucial to accurately inform ethical analyses of mental healthcare.

Participatory health research refers to a research paradigm that prioritizes the joint investigation of scientific and practical questions related to health and healthcare in a meaningful partnership between people with experiential knowledge and people with professional research backgrounds (17). It is rooted in grassroots organizing and social movements, for instance in Brazil and Colombia, where activists developed participatory action research with a strong focus on actions, emancipation and empowerment (18). Methods for participatory research were then increasingly considered for academic research.

The influential stage model of participation developed by Wright et al. (19) distinguishes nine levels of participation based on the amount of decision-making power allotted to people with experiential knowledge. The model goes back to the ‘ladder of citizen participation’ developed by Arnstein (20) and was subsequently adapted to the context of health research. The model describes a stage of non-participation which includes forms of instrumentalization. Here, people are only involved on a surface level, for instance to legitimize a project or to conform to regulations of a funding body, without having any possibility to influence the research. Many researchers are aware of both the risks and the ethical impermissibility of such forms of instrumentalization. According to a systematic review on patient engagement in healthcare, tokenism (i.e., the instrumentalizing involvement of marginalized groups) was identified as an overarching worry in various participatory projects (21). The stage model further identifies a preliminary stage of participation that includes the levels information, consultation, and inclusion. The next stage of participation involves the levels shared decision-making, partial delegation of decision-making authority, and full decision-making authority. Finally, the model describes a stage that goes beyond participation and is characterized by full decision-making power of people with experiential knowledge. Importantly, these different levels of participation can co-occur simultaneously within one project, for example in different phases of one project or within a project’s subprojects (22).

Several recommendations and practice guidelines for participatory health research exist (17, 23–25). In a good practice guidance explicitly developed for mental healthcare research, Schrank and Wallcraft (24) provide a detailed list of recommendations for researchers who want to engage in participatory research. The recommendations cover all phases of a participatory research project, including building a collaborative relationship with communities and user groups, identifying research priorities, undertaking a research project, disseminating and implementing research results. They also provide guidance on payment and budgeting. Important aspects include involving service users from the start of a project, being honest about one’s own goals and expectations (both towards oneself and towards communities and service users), and communicating clearly which levels of decision-making power can be achieved by people with experiential knowledge within the project. The authors stress the importance of planning sufficient resources to cover the extra time and funds required for meaningful participation and recommend communicating one’s research plan well in advance with the relevant departments at one’s research institution to reduce bureaucratic constraints.

In this article, we report our experiences with establishing a peer advisory board for an existing mental health ethics research group^2^. Patient, service user, or peer advisory boards are increasingly being implemented in academic research and service delivery (26–28). Such structures seem to be particularly well-suited to involve members of marginalized social groups in research processes. For instance, the project ‘PART-Beirat’ involves the establishment of two topic-specific advisory boards with people with lived experience of dementia and people with lived experience in forensic mental health care, respectively (27).

The SALUS research group (“The ethics of coercion: Striking a balance between autonomy, well-being and security in psychiatric practice”) was an independent and interdisciplinary research group at the Department of Psychiatry, Psychotherapy and Preventive Medicine and the Institute for Medical Ethics and History of Medicine at Ruhr University Bochum, Germany. The group received funding from the German Federal Ministry of Education and Research for a period of six years between 2018 and 2024. A trained peer support worker (AW) was part of the research group as a research assistant from the very beginning, but further forms of participatory research were not part of the initial project application. During the first project phase, SALUS group members came to recognize that this constituted a major constraint on their research and decided to establish a peer advisory board that allowed for different levels of participation (29).

In this article, we discuss the challenges, benefits, facilitators and lessons learned in relation to the implementation of a peer advisory board in the second project phase of an existing research project. Our analysis takes both the particularities of the model for participation we used and the field of empirical mental health ethics into account. By sharing our experiences, we wish to support other researchers aspiring to implement participatory research structures in their work. We will start by briefly describing the implementation process, and then continue to report the various forms of participation that emerged from the advisory board structure. Finally, we will discuss challenges, benefits, facilitators and lessons learned.

## 2 The SALUS peer advisory board

### 2.1 Development

A critical engagement with the SALUS research group’s potential to foster participation started with a discussion of participatory methods in a research colloquium. The SALUS group invited a person with experiential knowledge to the colloquium who later joined the peer advisory board (CS). This colloquium stimulated a critical reflection of previous research approaches among the SALUS group members, resulting in the decision to integrate further forms of participation in the research project. Subsequently, three SALUS group members (MF, SP, AW) held an online meeting with several service users recruited through the SALUS groups’ network to plan how this could be realized.

The SALUS group decided to use an online workshop on participatory research in mental healthcare with service users, relatives, people with lived experience, mental healthcare staff and researchers as a kick-off event. The workshop provided an overview of various participatory research projects and forms of participation. In break-out sessions, participants had the opportunity to discuss their own research interests in relation to several topics within the SALUS research group. At the end of the workshop, all participants were invited to get involved in the SALUS group’s research, for example by joining the peer advisory board. SALUS group members also encouraged attendants to develop peer-led research projects and offered to provide support and guidance. Several workshop participants were interested in joining the peer advisory board and contacted the SALUS group. A first online meeting with SALUS group members, service users and relatives was organized, during which a basic structure of the peer advisory board’s organization was developed. A SALUS group member formulated a first draft of an agreement detailing the aims, tasks, organization, and obligations of both SALUS group members and peer advisory board members. This draft was circulated among peer advisory board and SALUS group members, jointly discussed, revised based on the feedback received, and agreed upon by all parties involved. An English translation of the agreement can be found in the **Supplementary Material**.

### 2.2 The basic structure and organization of the board

The peer advisory board consisted of six women with a mean age of 50 years (ranging from 34 to 70). All members had completed or were completing formal peer support training, and four worked as peer support workers ("Genesungsbegleiter*innen"). Two members identified as people with experiential knowledge (“Psychiatrieerfahrene”), one as a relative, and three as members of both of these categories. In terms of professional status, two members were retired, four employed, one was enrolled in a part-time master’s program, and one was enrolled in a PhD program. Two members had completed professional training, three held university degrees, and one a PhD. One person worked as a mental healthcare researcher and two as mental healthcare staff. A research assistant in the SALUS group (AW) considered herself as a connecting link between the peer advisory board and the SALUS group itself. The peer advisory board was organized according to the following principles:

- Regularity: The research team organized four meetings per year.
- Accessibility: Meetings were held online, with one in-person meeting per year, to allow for the participation of people who lived in different regions of Germany and those with reduced mobility (for instance, due to job commitments or care work).
- Expenses: All expenses for participation in the in-person meetings (travel costs, accommodation) were covered by the SALUS group.
- Expense allowance: Peer advisory board members received an expense allowance for participation in in-person and online peer advisory board meetings.
- Transparency: SALUS group members prepared the peer advisory board meetings and presented a subproject on which they were currently working. The aims and contents of the meeting as well as an agenda were sent to peer advisory board members before each meeting.
- Accountability: A SALUS group member wrote a protocol for each peer advisory board meeting. Peer advisory board members received detailed meeting minutes and an anonymized short form of the meeting minutes for their personal use and their work in service users’ or relatives’ groups. The SALUS group member responsible for the meeting prepared a statement explaining how they incorporated the results of the meeting into their work.
- Confidentiality: All members agreed to maintain confidentiality regarding the personal information and experiences shared and the research content discussed in the meetings.
- The option of further involvement: To allow for flexibility and simultaneity of different forms of participation, SALUS group members provided peer advisory board members with a list of all subprojects within the research project. Peer advisory board members were invited to get involved in SALUS subprojects beyond their work in the peer advisory board. In this way, further collaborations evolved over time. We will highlight some of these in more detail below.

## 3 Examples of different forms of participation

### 3.1 Consultation on a study on informal coercion

In peer advisory board meetings, board members and SALUS group members jointly reflected on the group’s current research projects. This corresponds to the level of ‘consultation’ within the preliminary stage of participation according to Wright et al. (19). One such project was a qualitative interview study on treatment pressures and informal coercion. In this study, we investigated communicative strategies employed by relatives to influence service users’ decision-making processes and increase compliance with psychiatric treatment (30, 31). Study design, data collection, and the initial analysis of the data had already been completed. The peer advisory board was invited to jointly discuss the preliminary data analysis. During an in-person meeting, the responsible SALUS group members (CH and SP) presented the research design, the research questions and the preliminary coding framework. Peer advisory board members and SALUS group members read interview excerpts and analyzed them in small groups. All participants jointly discussed the SALUS group members’ interpretation of the excerpts and compared it to the analysis performed during the meeting. We discussed whether peer advisory board members found the categories comprehensible, how the categories related to their own experiences, and whether they missed any aspects in the SALUS group members’ analysis. The integration of experiential knowledge significantly improved the coding system.

### 3.2 Co-production

#### 3.2.1 Development of a template for a psychiatric advance directive

The development of a template for a psychiatric advance directive was a research project that involved the engagement of peer advisory board members on multiple levels. In this project, we developed a template for a document that allows service users to state their wishes for mental health crises in advance. Initially, SALUS group members (ASG in consultation with EB and MS) developed a template prototype based on two systematic reviews conducted by the SALUS group (32, 33). This prototype was then evaluated in a focus group study with several stakeholder groups (service users, relatives, professionals, legal guardians, and peer support workers). The template prototype and the study design were presented in a peer advisory board meeting, and peer advisory board members provided feedback both during the meeting and via email. After this meeting, one peer advisory board member (IrH) decided to join the core research team for this project. This member subsequently participated in focus groups, data analysis sessions and regular project meetings. The feedback obtained in the focus groups was discussed within the core team until consensus was reached, and the template was revised accordingly (Gaillard et al.^3^). This involvement corresponds to the level of ‘shared-decision making’ within the stage of participation according to Wright et al. (19). Results of the focus groups and the revised template were presented in another peer advisory board meeting to obtain additional feedback, which was integrated into the template.

#### 3.2.2 Co-writing of academic articles

Members of the peer advisory board and the SALUS group jointly developed and co-wrote two articles. The first was a reflection article on the collaboration between the SALUS researchers and the peer advisory board (29). In this article, we discussed the opportunities and challenges of a peer advisory board, as well as the development of our expectations and concerns over two years of collaboration.

The second was a research article on dual-role dilemmas in psychiatry. Dual-role dilemmas refer to the ethical obligations of mental healthcare workers to simultaneously support mental health service users and to protect third parties from harm (e.g., other people on the ward or in the community) (Efkemann et al.^4^). Zinkler and von Peter (34) have argued that psychiatry should solve these dilemmas by focusing solely on supporting patients. Two SALUS group members (SE and SP) discussed this argument with peer advisory board members based on their phenomenological, emotional and practical knowledge on coercion in mental healthcare. All authors contributed their evaluations of psychiatric practices based on their own experiences and positions, sometimes taking diametrically opposite positions.

We co-wrote both articles using ‘shared-decision making’ within the participation stage according to Wright et al. (19). We used a similar method for both articles. All authors regularly met online to discuss the research question and the article outline, as well as to share their perspectives and emotions on the topic. These meetings were a crucial part of the writing process. Encouraging moderation created an atmosphere where everyone felt comfortable to express their point of view, even when people considered their point of view controversial. While this also led to tension and irritation, the debate stimulated critical reflection, and many participants reported changing their point of view during this process. In the writing process, all authors contributed notes or formulated paragraphs that SALUS group members subsequently integrated into the manuscript draft. SALUS group members then revised the draft based on several rounds of feedback in an iterative process, until everyone felt that their position was adequately represented in the article.

These two articles were co-written with all peer advisory board members. Additionally, individual peer advisory board members also joined other article projects within the SALUS project, e.g. a book chapter on police operations (Efkemann et al.^5^).

#### 3.2.3 Joint preparation of presentations and scientific events

Results from the joint scientific activities were also prepared for presentation at various national conferences. For instance, some peer advisory board members and SALUS group members jointly organized symposia at the annual congress of the German Association for Psychiatry, Psychotherapy and Psychosomatics (DGPPN). In one symposium, members of the peer advisory board presented their positions on informal coercion in mental healthcare. In another, IrH spoke about her experiences as a peer researcher in the development of a template for a psychiatric advance directive.

Moreover, all peer advisory board members participated in planning the two-day final conference of the SALUS project. In several joint online meetings, we decided to include the perspectives of service users, relatives and mental health professionals in the individual conference sessions. For these perspectives, we did not only recruit speakers from among ourselves, but also invited external stakeholders via peer advisory members’ networks. Furthermore, we organized a session on participatory research in which both peer advisory board members and SALUS group members shared their experiences on their collaboration.

### 3.3 User-led research

#### 3.3.1 Developing an auto-reflective account of recovery

During peer advisory board meetings, we repeatedly engaged with the topic of recovery. This resulted in one peer advisory board member’s (UL) wish to reflect on her own crisis and work on her recovery story. She had already been interested in the concept of recovery before joining the peer advisory board and the encouragement of one SALUS group member (MF) helped her to develop her project in more detail. The initial idea was to co-write an article on well-being and recovery from various perspectives: her own as an expert by experience, that of a psychiatrist, and that of a mental health ethicist. During the course of the project, however, UL realized that she would prefer to use her own voice to compose her personal story. From then on, she pursued the project alone and wrote a single-authored article, receiving feedback and guidance for publication by one SALUS group member (MF) according to her own needs (Lux^6^). According to the stage model of participation by Wright et al. (19), this project goes beyond participation as UL had full decision-making power.

## 4 Discussion

### 4.1 Challenges

SALUS group members faced several organizational and fundamental challenges in relation to building the peer advisory board and our joint work. Regarding organizational challenges, SALUS group members found it difficult to combine their aspirations of participation with existing bureaucratic standards set by funding bodies or universities. Existing recommendations stress the need to contact the relevant departments, such as the finance department of the university, in advance (24). A barrier to this is that typical research grant applications require submitting a detailed financial plan before beginning the research project. Consequently, changing the allocation of funding over the course of the funding period proved challenging due to the rigid bureaucratic procedures already in place. This was detrimental to the involvement of peer advisory board members in decisions on how to spend research funding and limited the extent of power sharing possible within our project.

As often noted, organizing meaningful participation in research takes time (24, 27, 28). For SALUS group members, the organization of the peer advisory board meetings, writing protocols and statements required a significant time investment. Likewise, peer advisory board members sometimes found it challenging to find the time and resources to be substantially involved in different projects. Some subprojects, such as creating publications, required more time than the peer advisory board meetings themselves. However, most peer advisory board members only received financial compensation for the meetings, not for the extra time spent on these subprojects (with the exception of the joint development of the template for a psychiatric advance directive). Additional compensation for all emerging projects would have been necessary (24) but was not possible to obtain due to the bureaucratic challenges mentioned earlier. These constraints narrowed the scope of possible members for a peer advisory board, as only people with sufficient financial and time resources could choose to get involved. Finally, some peer advisory board members experienced the uncertainty about how the collaboration would continue after the end of the SALUS group’s funding period as stressful.

One fundamental challenge concerned the composition of the peer advisory board, which was homogenous in some respects: it predominantly consisted of female members with a higher academic education who had completed peer support training. However, a person’s intersectional social position and associated experiences of discrimination can lead to specific challenges in mental healthcare. Structural discrimination is essential for ethical analyses (35), especially in the ethics of coercion (36). Therefore, it would have been preferable if peer advisory board members had represented a broader range of genders, more diverse educational and class backgrounds, and included racialized people. This is particularly important as failing to sufficiently include members of marginalized groups in participatory research can risk reproducing discrimination (37).

As another challenge, peer advisory board members noted that they often contributed personal or intimate information as part of their role, whereas SALUS group members could retain a more distant ‘professional’ role (29). While we consider having created a space in which such personal information could be shared as an accomplishment, this automatically created an imbalance in perceived personal risks and burdens between SALUS group members and peer advisory board members. In reflecting on our joint work while writing this article, we noted that we did not succeed in fully overcoming the division between both groups.

Some of us found that the structural power imbalances underlying this division were not sufficiently acknowledged and discussed within the group, even though power imbalances constitute a well-known barrier to participatory research (38, 39). Hierarchies were further exacerbated by organizational factors, such as agenda setting by SALUS group members, the impossibility of compensating peer advisory board members for some forms of work (e.g., writing articles), and limited flexibility in adapting the SALUS group’s budget to the research priorities of peer advisory board members. Such power imbalances within a group may be detrimental to open and equal communication and exchange, impacting the possibility of people with relatively less power to speak up or to be heard. Such exclusions, sometimes referred to as epistemic injustices (40), are particularly common in the context of mental healthcare (3, 7, 10, 41). In our project, the SALUS group failed to ensure full transparency about the involvement of peer advisory board members in SALUS sub-projects, which also led to knowledge asymmetries among peer advisory board members.

### 4.2 Benefits

We identified several benefits of having established the peer advisory board and of our joint work, both for peer advisory board members and for SALUS group members.

First, the peer advisory board provided space for the personal development and growth of its members by offering flexible opportunities to experiment with different forms of engagement in research. Participants’ personal development and empowerment is an important goal of participatory research approaches (17). Especially the possibility to opt in and out of projects allowed peer advisory board members to experiment with research while staying in control of their involvement. The flexibility of the peer advisory board format supported individual capacity-building, which is considered a prerequisite for establishing sustainable models of service user participation in research and service development (4). Peer advisory board members not only acquired knowledge on topics within mental health ethics, but also developed new capacities and skills, such as how to write an academic article, develop a research question, or argue for a controversial position, an experience also shared by participants in community-based participatory research (42).

Participating in research projects according to their own interests and preferred levels of involvement allowed some members to increasingly assume the role of a researcher on their own terms – to develop their own questions, follow their intuitions, or find their position in a debate. Many peer advisory board members experienced this as empowering. Peer advisory board members also reported transferring capacities and knowledge acquired within the peer advisory board to other contexts, such as user groups, mental health initiatives, and other research projects. Peer advisory board members felt like, and in fact were, multipliers of the research developed within the SALUS project.

For SALUS group members, the peer advisory board provided a space to explore and discuss their research with people with experiential knowledge. In the literature, mutual learning, personal growth, more in-depth understanding of qualitative data, and increased awareness of different vantage points are described as important benefits of participatory research from the perspective of researchers without experiential knowledge (38, 39). SALUS group members largely shared these experiences. In terms of mutual learning and enhanced research, the experiential knowledge brought in by service users and relatives was an invaluable resource for developing ethical recommendations acceptable for people with different experiential backgrounds. On multiple occasions, this experiential knowledge helped SALUS group members to uncover knowledge gaps and biases. Additionally, given that they work on contested topics within mental health ethics, SALUS group members welcomed the possibility to experience accountability for their research and to justify their approach to a contested topic in a critical but charitable setting. This helped SALUS group members to ensure their research was relevant to and acceptable for the people affected by the research.

Our discussion of the identified benefits highlights that measuring the impact of participatory research exceeds the scope of standard evaluations. Friesen et al. (4) argue that when evaluating the impact of participation, many researchers go back to measurable effects, such as influence on patient recruitment, the rigor of analyses, or the dissemination of results (43). However, such analyses may distract from *ethical* reasons to involve people with experiential knowledge that arise from their history of marginalization and powerlessness within the psychiatric system. These ethical reasons are salient irrespective of any instrumental goals of participation (4). Following Friesen et al. (4), we would like to stress that for us, the most important benefits of establishing the peer advisory board were personal development and empowerment, community and individual capacity-building, and gaining experience with power sharing. While these factors are difficult to ‘measure’ or ‘objectify’, qualitative research methods from the social sciences, e.g. interview and focus group studies, may be apt to capture such promotion of ethical values, as demonstrated in the research accompanying the implementation of the peer advisory boards within the ‘PART-Beirat’ project (27).

### 4.3 Facilitators

Reflecting on our experiences, we identified several facilitators that enabled the establishment of the peer advisory board which allowed for flexible forms of participation.

First, employing a person with lived experience as a research assistant in the preparation of establishing the peer advisory board was very important. This influenced the research focus and the discourse within the research team from the beginning of the SALUS project. For the SALUS group, it was helpful to have team sessions in which team members reflected on why exactly they wanted to involve people with lived experience, which concessions to their customary style of doing academic work they were willing to make, and which additional tasks and responsibilities they were willing to assume. These meetings made SALUS group members realize that meaningfully involving service users and relatives would allow them to better tailor their research to the research needs of the people affected and motivated them to invest time and resources in doing so.

Second, prior to the collaboration, we arranged a preparatory meeting of the peer advisory board to make our expectations of the collaboration transparent. To this end, we co-created and found consensus on a framework of mutual expectations, which included a distribution of responsibilities in the form of a more official agreement. Similarly, other community advisory boards found it important to clarify roles and responsibilities at the beginning of a new collaboration to ensure accountability and transparency (28). Also, developing a functional infrastructure for communication and information sharing helped to increase transparency. Finally, investing effort in the detailed preparation of meetings (e.g., with meeting invitations, agendas, minutes, and an established format for input and discussion) strengthened a sense of commitment and trustworthiness in the collaboration.

The long-term funding of the SALUS project enabled the SALUS group to implement forms of participation even after the project had already begun. After initiating the peer advisory board, the perspective of having at least three years of cooperation offered us time to get to know each other, find a shared language, and foster understanding. This time frame facilitated the successive development of mutual trust, which is considered an important basis for participatory research (44).

This increasingly allowed collaboration on more complex and abstract projects. Mental healthcare ethics involves philosophical research projects, which have a higher threshold to participation given that they require more abstraction and prior conceptual knowledge. Having co-written a reflection article (29) before working on a reflection of an ethical argument (Efkemann et al.^4^) allowed for the development of philosophical capacity in the peer advisory board over time. Even though no peer advisory board member contributed to the development of a philosophical argument published by the SALUS group as a co-author so far, we believe that based on our joint capacity building, this form of collaboration would now be possible.

Another facilitator was the peer advisory board’s structure. Its flexibility allowed for different levels of participation to co-occur, a strength also noted by other patient advisory boards in healthcare (e.g., 45). The peer advisory board members were invited to adapt their level of participation according to their current situation, interests, and preferences. Such a flexible model was also positively evaluated by Weinstein et al. (28), who also organized consultations on existing research while allowing for new projects to emerge during the collaboration in their community advisory board. The board’s flexible structure enabled people with diverse personal and professional situations to get involved in the peer advisory board on their own terms. The effort to implement such flexibility also encouraged SALUS group members to adapt their projects, making their research more dynamic. The “work in progress” character of participation in the context of advisory board structures is also highlighted in the literature (45).

An important facilitator of participation was respectful, open, and authentic communication between SALUS group members and the peer advisory board. Many peer advisory board members experienced the working environment provided by the SALUS group as welcoming and supportive, offering guidance and feedback where necessary, but also allowing for independent exploration of an area of interest. The fact that some peer advisory board members also had experience with academic research and that some SALUS group members had personal experience with using mental health services facilitated mutual understanding and trust, especially when SALUS group members shared their own experiences of mental distress. Peer advisory board members had very diverse experiences with mental healthcare, and held differing background beliefs about psychiatry and psychiatric treatment options. This allowed for balanced and precise discussions. In addition, having a ‘double qualification’ (i.e., expertise by experience and academic expertise) made several of the peer advisory board members uniquely qualified to engage in knowledge sharing and capacity building among their peers in the community, forming a bridge between academia and the community.

### 4.4 Lessons learned and ways forward

We now describe some final lessons that we learned while carrying out participatory mental health ethics research. First, in terms of diversity, we would try to increase our efforts to reach out to marginalized people in the future. While marginalized communities are sometimes referred to as “hard-to-reach,” Kalathil (46) stresses that using such terms sometimes masks that researchers have not put sufficient effort into reaching out to marginalized communities. Possible avenues would be advertisement through additional communication channels or social media, or by actively approaching people from backgrounds that are not yet represented in the research team.

Second, we would organize specific training to prepare and accompany the participatory research, as demonstrated in other advisory boards, e.g. in the ‘PART-Beirat’ project (27). As the National Institute for Health Research elaborates, such training, if tailored to the individual project and participants, can help *all* parties to strengthen their knowledge and skills for participatory research (47). Importantly, when planning such training, it is important not to assume that people with professional research backgrounds are sufficiently trained while people with experiential knowledge lack the relevant competencies. By contrast, *all* people involved have individual skills, knowledge and expertise that can be adapted for the respective project by receiving suitable training. Such training could provide communication training, a general introduction to research, as well as an overview of the relevant research field. Such training could help all participants to define their own role in the project more clearly from the beginning. The National Institute for Health Research recommends involving people with lived experience and research experience in participatory projects in the preparation and delivery of such training (47).

Third, to better address power and information asymmetries in future projects, we would schedule regular team meetings to reflect on and openly discuss power imbalances, as these persisted within our group in spite of everyone’s best intentions. Friesen (3, 128 [italics in original]) suggests the following reflection questions that we would use in the future: “*Is power truly distributed? Is the community involved the appropriate one? Are those involved diverse or merely an agreeable subset of patients? Are there opportunities to ask questions and identify assumptions or biases in the methodology or other aspects of the research?*” Additionally, we would also organize meetings among peer advisory board members (without SALUS group members) to reflect on team dynamics, exchange their experiences with the research process, increase joint awareness of possible imbalances and to mitigate these. Also, including a person with experiential knowledge who is not part of the project for external peer mentoring, as recommended by the National Institute for Health Research, appears helpful for addressing such power imbalances (47).

Finally, we see several improvements that funding agencies, universities and academic journals could implement in order to reduce barriers and make participation possible. Funding agencies could expand formats tailored to the specificities of participatory research. For instance, formats that provide funding to enable academic researchers and experts by experience to jointly prepare a funding application for a research project would be particularly helpful to ensure that people with lived experience participate in setting research priorities and designing research projects. If possible, universities should strive to make bureaucratic structures more flexible to enable the formal involvement of people with lived experience (e.g., as employees) and guarantee adequate financial compensation for their work. This compensation should go beyond a reimbursement of out-of-pocket expenses and include financial compensation for invested time or delivered services. Payment models could be time- or honorarium-based and the height of the compensation should be based on the nature of the research role or activity, the expertise of the co-researcher, the required time commitment and local payment standards. Guidance for the active involvement of people with lived experience in research is currently being developed, for example by the National Institute for Care and Research (48). Finally, academic journals could be more open to non-traditional ways of academic writing and forms of communicating scientific knowledge. A way to start would be to create special article types for participatory research studies but ultimately, standards for original articles should be adjusted to accommodate for participatory research.

## 5 Conclusion

In this article, we have discussed the challenges, benefits and facilitators of implementing a peer advisory board within an existing mental health ethics group. Establishing a peer advisory board after the project’s beginning proved challenging yet feasible, especially because researchers were committed to invest time and energy. We experienced the collaboration as an invaluable opportunity to better understand the intricate ethical issues in mental healthcare. Participatory research has many important benefits that, from our perspective, outweigh the time and effort required. Our experiences also demonstrate, however, that institutional changes in academic research are necessary to make the broader implementation of participatory research possible.

At the same time, institutional and structural barriers should not lead academic researchers to shy away from attempting to establish peer advisory boards for their research projects. Despite the challenges we faced, the SALUS peer advisory board enabled SALUS group members to discuss their research with people with lived experience, to set new research priorities, to promote community outreach and to increase their accountability for their research. For peer advisory members, participation in the SALUS peer advisory board led to personal development and growth as well as a sense of both individual and community empowerment. Notably, the SALUS peer advisory board proved to be a gateway to deeper levels of involvement and participatory research, including the co-production of an ethical intervention in the field of mental health (i.e., the template for a psychiatric advance directive), the co-authoring of academic articles and the joint presentation of research findings at scientific conferences. Our experience thus reminds us that the challenges of participatory research should not be seen as mere obstacles but rather as opportunities to reimagine academic research in the field of mental health ethics as a collaborative and empowering pursuit.

## Footnotes

1 Helmchen H, Gather J, Sartorius N eds. Ethics in Psychiatry. 2nd Edition. (Heidelberg, Berlin: Springer). (Forthcoming).

2 In the following, we use ‘we’ when taking the perspective of all authors, and ‘peer advisory board members’ and ‘SALUS group members’ to refer to members of these two groups, respectively.

3 Gaillard AS, Gather J, Haferkemper I, Vollmann J, Potthoff S, Scholten M, et al. Developing a Template for a Psychiatric Advance Directive: A Qualitative Study with Key Stakeholders (Submitted).

4 Efkemann S, Haferkemper I, Heuer I, Lux L, Spiegel S, Stefaniak C, et al. Die Doppelfunktion der Psychiatrie: Unterstützungs- und Schutzauftrag – (k)ein Widerspruch? Eine Reflexion von Betroffenen, Angehörigen und Forschenden zu Ethikund Zwang in der Psychiatrie. Recht und Psychiatrie. (Forthcoming).

5 Efkemann S, Heuer I, Gather J. Der polizeiliche Kontakt zu Menschen in psychischen Krisen unter Berücksichtigung der Perspektive von Personen mit eigener psychischer Krisenerfahrung und Professionellen aus der Psychiatrie. In: Staller M, Körner S, Zaiser B, editors. Polizei und Menschen in Psychischen Krisen. Heidelberg, Berlin: Springer. (Forthcoming).

6 Lux U. Mein ganz persönliches Recovery – Krankheit als Weg zur Gesundung. (In press).
