# Supplementary material for: Establishing a peer advisory board in a mental health ethics research group – challenges, benefits, facilitators and lessons learned
Source: Front Psychiatry. 2025 Feb 28;16:1516996. doi: 10.3389/fpsyt.2025.1516996 (PMC11907373; doi:10.3389/fpsyt.2025.1516996)
Supplement: Supplementary file 1 [file DataSheet1.pdf]

Agreement for the service users/people with lived experience/relatives advisory board[1] of the BMBF research group SALUS

§1 Objective

The objective of the peer advisory board is to contribute to better integrating the perspectives and experiences of psychiatric service users/people with lived experience and their relatives[2] into the research conducted by the SALUS group.

The aim is to promote an open dialogue between psychiatric service users, their relatives, and members of the SALUS group.

The goal is to contribute to the improvement of research. The participation of psychiatric service users/people with lived experience and their relatives[3] in research on social and medical practices is essential to reflect on implicit assumptions and different evaluations of study results during the planning and evaluation of scientific research.

In addition, the advisory board's guidance is intended to broaden a power-critical perspective on the SALUS group's research.

A further aim is to provide the members of the advisory board with opportunities for new areas of agency in the sense of *empowerment*. This includes the following possibilities: gaining insights into medical ethics research; presenting and using results of the SALUS group's research in their communities and self-organized support groups and for their own goals, such as the improvement of care for people with mental illness or the support of their relatives, participation in the journal club and workshops of the research group.

§2 Tasks

The advisory board members' tasks are:

- General consultation of the SALUS research group regarding content
- Concrete feedback on research processes regarding the research question, planning, recruitment strategy, data collection, data analysis and dissemination of results
- In addition, further tasks are possible if members are interested, e.g. concrete feedback on scientific articles, recommendations and manuals for psychiatric practice, presentation of results at conferences or other events, collaborating on data analysis, co-writing of articles and manuals.

§3 Structure

The advisory board meets regularly every three months on a fixed date. The kick-off meeting takes place in person in Bochum. Subsequently, at least one meeting per year takes place in person. The other meetings are held digitally or in person, in consultation with the members. Digital meetings last 1.5 hours and in person meetings last 3 hours.

---

[1] In German: „Betroffenen/Nutzer*innen/Psychiatrieerfahrenen/Angehörigen Beirat"
[2] In German: „Betroffenen/Nutzer*innen/Psychiatrieerfahrenen und deren Angehörige"
[3] In German: „Betroffenen/Nutzer*innen/Psychiatrieerfahrenen und deren Angehörige"

Members of the research group attend each meeting to present a project or question that they would like to discuss and reflect on together with the advisory board. The advisory board members discuss the project together with the SALUS group members and give their recommendations, objections, and evaluations. It is not necessary for peer advisory board members to reach consensus.

A research assistant of the SALUS group is present and records the discussion, the objections and recommendations of the advisory board in anonymized form. A research assistant of the SALUS group is responsible for organizing the meetings.

§4 Members

- The advisory board consists of eight members.
- The members should represent the groups of persons included in the group's research. This includes people with severe mental illness and their relatives, as well as people with experience in forensic psychiatry and/or experiences of multiple discrimination.
- The composition of the advisory board should be as diverse as possible in terms of educational background, experiences of discrimination, gender, age and illnesses.

§5 Nomination and appointment

The members of the advisory board are appointed by the members of the research group for at least one year, and at most until the end of the SALUS project duration (10/2024). The duration of the appointment depends on the wishes of the respective advisory board member.

If a member leaves prematurely, another member can be appointed for the duration of the term of office of the advisory board. In justified cases, individual members may be dismissed from the advisory board prematurely. A premature dismissal and a new appointment both require a 2/3 approval of the advisory board as well as a 2/3 approval of the research group. The research group includes all academic staff and research assistants.

If they wish so, the advisory board members are named on the SALUS website.

§6 Obligations

The advisory board and research group members commit to mutual confidentiality and reliability.

Advisory board:

- All members should be willing and able to regularly attend the meetings of the advisory board.
- All members commit themselves to maintain confidentiality towards people outside of the advisory board and the research group regarding the research content discussed in the meetings as well as personal information shared by the participants.

Research group:

- All subprojects of the SALUS group must be presented and discussed at least once in an advisory board meeting.

- All SALUS employees commit themselves to present the projects they are involved in to the advisory board at least once.
- The SALUS group commits itself to transparency regarding how it incorporates the advisory board's objections and recommendations into its work. (1) The researchers who have introduced a question in the advisory board write a short statement of accountability afterwards. The minutes and the statement are shared with all members of the advisory board and the SALUS research group within four weeks after the meeting. (2) The consultation with the advisory board is described in the methods section of publications and the implementation of the advice is explained. (3) After the completion of a sub-project, the minutes and the statement are published on the SALUS website.
- All SALUS employees commit themselves to confidentiality regarding the contents discussed in the meetings towards people outside of the advisory board and the research group.

§7 Reimbursement of expenses

Each member receives an expense allowance of €20 per online meeting and €40 per meeting in person. In addition, travel costs for in person meetings are reimbursed.

§8 Entry into force

This agreement enters into force after the advisory board members' approval with the decision of the SALUS group.
